# Co-administration of Apelin and T4 Protects Inotropic and Chronotropic Changes Occurring in Hypothyroid Rats

**DOI:** 10.5935/abc.20150086

**Published:** 2015-09

**Authors:** Zahra Akhondali, Mohammad Badavi, Mahin Dianat, Farzaneh Faraji

**Affiliations:** Physiology Research Center and Department of Physiology, Faculty of Medicine, Ahvaz Jundishapur University of Medical Sciences, Ahvaz – Iran

**Keywords:** Hypothyroidism / blood, Thyroid Hormones / blood, Thyroxine / therapeutic use, Cardiotonic Agents, Rats

## Abstract

**Background:**

One of the most important thyroid hormone targets is the cardiovascular system. Hemodynamic changes, such as decreased resting heart rate (HR), myocardial contractility, and cardiac output, and increased diastolic pressure and systemic vascular resistance, have been observed in hypothyroid patients. Moreover, in these patients, ECG changes include sinus bradycardia and low voltage complexes (P waves or QRS complexes).

**Objective:**

This study aimed at evaluating the prophylactic effect of apelin on HR changes and QRS voltage that occur in propylthiouracil (PTU)-induced hypothyroid rats.

**Method:**

In this study, 48 adult male Wistar rats weighing 170-235g were randomly divided into 6 groups: Control group (normal saline ip injection + tap water gavage); P group (PTU 0.05%, in drinking water); A group (apelin 200 µg.kg^-1^.day^-1^, ip); PA group [co-administration of PTU and apelin]; PT group [co-administration of PTU + T4 (0.2 mg/g per day, gavage)]; and PAT group (co-administration of PTU, apelin and T4). All experiments were performed for 28 consecutive days, and then the animals were anesthetized with an ip injection of ketamine (80 mg/kg) and xylazine (12 mg/kg). Lead II electrocardiogram was recorded to calculate HR and QRS voltage.

**Results:**

Heart rate and QRS voltage increased more significantly in the hypothyroid group that consumed both apelin and T4 (201 ± 4 beat/min, 0.71 ± 0.02 mv vs. hypothyroid 145 ± 9 beat/min, 0.563 ± 0.015 mv; respectively).

**Conclusion:**

The co-administration of apelin and T4 showed a protective effect on QRS voltage and HR in PTU‑induced hypothyroid rats.

## Introduction

Thyroid hormone is necessary to regulate metabolic rate^1^. Thyroid hormone also has an effect on the cardiovascular function, in which a minimal decrease of circulating thyroid hormones may cause cardiovascular dysfunction^2^. In patients with overt hypothyroidism, lack of thyroxin (T4) feedback leads to TSH levels higher than those in healthy individuals, whereas in milder or subclinical hypothyroidism, T4 and triiodothyronine (T3) levels are normal, but TSH levels are higher than in healthy people^3^. As a result of the loss of thyroid hormones, some structural and morphological changes occur in cardiac cells causing changes in heart hemodynamic characteristics. Decreased resting heart rate (HR), cardiac output, heart contractility and stroke volume, as well as increased systemic vascular resistance and diastolic pressure have all been identified in hypothyroidism. Furthermore, bradycardia, narrow pulse pressure, voltage reduction and cardiac block have also been observed^4-6^. Heart rate variability and heart rate turbulence are the criteria of cardiovascular autonomic function that change in hypothyroid patients^5,7^. One of the criteria of cardiac contractility is the QRS amplitude.

In hypothyroid patients, ECG manifestations, such as sinus bradycardia, low voltage complexes (small P waves or QRS complexes), prolonged PR and QT intervals, and flattened or inverted T waves have been observed^8^. In addition, pericardial effusion, which could affect ECG, has been shown in up to 30% of hypothyroid patients^8^.

Moreover, angina and myocardial infarction have been observed approximately in 1% of the general population, and 4% of individuals 60 years and older are prescribed long-term T4^9,10^.

Apelin is an endogenous ligand that is expressed throughout a number of tissues such as heart, brain, liver, skeletal muscle and kidney. Apelin acts through the APJ receptor, a G protein-coupled receptor, and shares similarities with the angiotensin II-angiotensin II type 1 receptor pathway^11,12^. Apelin causes endothelium-dependent vessel vasodilation through eNOS activation, and promotes NO release. Furthermore, apelin and APJ receptor have an effective role in the development of cardiac cells^13^. This endogenous ligand, which affects myocardial cells, causes an increase in cardiac contraction^14,15^. It has been reported that apelin has an inotropic effect on heart^16^.

Since the contraction of heart muscle is associated with QRS voltage, and considering that the QRS complex voltage and HR are reduced in hypothyroid patients, the aim of the present study is to evaluate the protective effect of apelin on the inotropic and chronotropic changes that occur in the absence of thyroid hormones.

## Methods

### Materials

Ketamine and xylazine were purchased from Alfas Co. (Holland). Propylthiouracil (PTU) and T4 were obtained from Sigma-Aldrich Co. (USA), and apelin from Cayman Chemical Co. (USA). PTU was dissolved in drinking water (0.05%), and apelin, at the dose of 200 µ g.kg^-1^.day^-1^, was dissolved in normal saline and injected intraperitoneally (ip). L-thyroxin was dissolved at first in 0.1 normal NaOH and then diluted with tap water to the desired concentration (0.2 mg/g per day).

### Animal treatment

Forty-eight male Wistar rats (170-235 g) were housed in standard conditions (22+2°C, 12/12 h light-dark cycle) with free access to standard rat chow diet (Pars Co. IR) and tap water ad libitum. All procedures were performed in accordance with the standards for animal care, established and approved by the Research Committee of the Ahwaz Jundishapour University of Medical Sciences, Ahwaz, Iran.

The rats were divided into six groups of eight animals each: Control group; hypothyroid group (P) [treated with PTU (0.05%)]; A group [treated with apelin (200 µ g.kg^-1^.day^-1^), ip]; PA group [co-administration of PTU and apelin]; PT group [co-administration of PTU and T4, gavage]; and PAT group [co-administration of PTU, apelin and T4]^17-19^. The treatment period in each experiment was four weeks.

Body weight was assessed every week and the serum levels of T4 and TSH were assayed at the end of experiments. After the procedures, the animals were anesthetized with an ip injection of ketamine (80 mg/kg) and xylazine (12 mg/kg). Rectal temperature was continuously monitored and maintained within 37-38°C using a heat pad and heat lamp. Lead II electrocardiogram was recorded to calculate HR and QRS voltage (PowerLab, ADInstruments, Australia) as follows: electrodes consisting of 26-gauge needles were placed subcutaneously for 1 cm at the xiphoid cartilage (positive electrode), right shoulder (negative), and left shoulder (reference). Electrodes were connected to a Bioamp amplifier (ADInstruments, Australia) and were digitalized through an A/D converter PowerLab 8sp (ADInstruments, Australia). Digital recordings were analyzed with Chart software for Windows 7 (ADInstruments, Australia). Events were registered to 4 K/s and were filtered to 60 Hz^20^. The ECG was calibrated for 25 mm/s with a sensitivity of 10 mm = 10 mV. ECG recordings were obtained for five minutes. The QRS complex voltage (in mV), to assess inotropic changes, was measured manually as the sum of absolute voltages of any positive or negative deflection. All calculations were made on the average of five QRS complexes. The HR, to assess chronotropic changes, was derived from the ECG signal.

### Statistical analysis

Statistical analysis was performed using SPSS and the data are expressed as the mean ± SEM. Comparisons were made by using one-way analyses of variance (ANOVA), which was followed by a Least Significant Difference (LSD) test. p < 0.05 was considered statistically significant.

## Results

### Serum TSH and T4 levels

In the hypothyroid rats, the serum levels of TSH increased, while T4 levels decreased significantly as compared with those of the control group (p < 0.01, Table 1). These values indicated that hypothyroidism induction by PTU was successful. Apelin administration with PTU prevented the rising of TSH and T4 levels. In addition, increased T4 and decreased TSH levels were shown in the PT group as compared with the hypothyroid group (p < 0.01). Although the co-administration of these three drugs prevented the rising of TSH and the decline of T4 levels, there was still a significant difference with the control group (p < 0.01).

**Table 1:** Comparison of T4 and TSH hormone level in different groups

**Groups**	**T4 (nmol/L)**	**TSH (μlU/mL)**
CO	76.75±5	1.32±0.3
P	24.49±3.1**	12.02±2.8**
A	87.59±5.27††	0.84±0.3††
PA	16.49±2.14**€€	5.09±0.57††
PT	95.50±0.25**††€€	8.78±1.56**
PAT	50.26±0.11**††	9.49±1.88**

CO: control group; P: PTU-treated group; A: Intact animals group that received apelin; PA: receiving PTU and apelin at the same time; PT: receiving PTU and T4at the same time; PAT: receiving PTU, apelin and T4 at the same time. *p < 0.05;

** p < 0.01 compared to the control group; †p < 0.05;

†† p < 0.01 compared to the P group,

€€ p < 0.01 compare to the PAT group (mean±SEM, n=8, one-way ANOVA followed by LSD test).

### Body weight

As expected, body weight in the control group increased significantly; however, in the hypothyroid group, it was reduced during the four weeks of PTU administration (p < 0.01; Table 2). On the other hand, in the euthyroid rats, administration of apelin led to a significant increase in body weight (p < 0.01). Furthermore, compared with the control and hypothyroid groups, co-administration of PTU and apelin reduced body weight in intact animals (p < 0.01, p < 0.05). Although T4 hormone therapy replacement with apelin in these rats could prevent weight loss, there is still a significant difference with the control group (p < 0.05). However, with combination of these three drugs, the weight gain could match that of the control group. Regarding the heart weight changes, it should be mentioned that although the ratio of heart weight to body weight did not change in the hypothyroid group, it was enhanced by apelin in the euthyroid group (p < 0.01). Moreover, this ratio did not change in the other groups (Table 2).

**Table 2:** Comparison of body weight and heart weight in different groups

**Groups**	**BW1 (g)**	**BW2 (g)**	**Heart weight(g)**	**Gain (g)**	**% changes'**	**HW/BW (g)**
CO	232±5	248±5	0.82±0.04	16±2	7	0.32±0.01
P	224±7	219±6	0.75±0.03	-5±3**	-2**	0.35±0.01
A	169±3	200±5	0.80±0.03	31±3**††	19**††	0.39±0.01**†
PA	169±4	156±3	0.51±0.01**††€	-13±3**†€€	_-_7**†€€	0.33±0.01
PT	206±4	209±6	0.72±0.02**€	3±3*	1*	0.34±0.01
PAT	176±5	183±7	0.62±0.03**††	11±4††	6††	0.35±0.01

CO: control group; P: PTU-treated group; A: Intact animals group that received apelin; PA: receiving PTU and apelin at the same time; PT: receiving PTU and T4at the same time; PAT: receiving PTU, apelin and T4 at the same time.

BW1: body weight at the beginning of experiment; BW2: body weight at the end of experiment HW: heart weight; HW/BW: ratio of heart weight to body weight. a: percentage of variation compared to the initial body weight

* p < 0.05;

** p < 0.01 compared to the control group;

† p < 0.05,

†† p < 0.01 compared to the P group,

€ p < 0.05,

€€ p < 0.01 compare to the PAT group (mean±SEM, n=8, one-way ANOVA followed by LSD test).

### Heart rate

**Figure 1:**
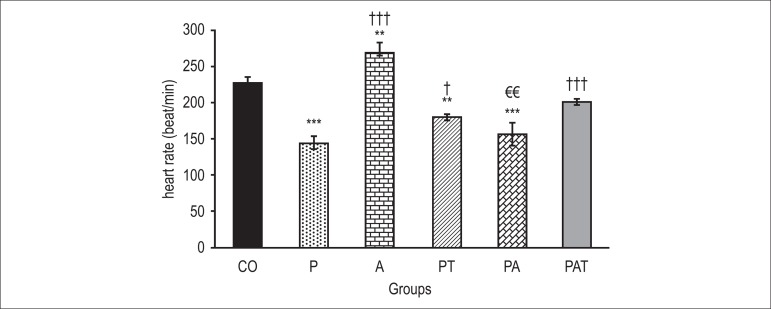
Comparison of heart rate in various groups. Data are expressed as mean±SEM. The analysis of data was done by using one-way ANOVA followed by LSD test. **p < 0.01, ***p < 0.001 compared to the control; †p < 0.05, †††p < 0.001 compared with the hypothyroid group; €€ p < 0.01 compared with the PAT group. CO: control group; P: PTU-treated group; A: Intact animals group that received apelin; PA: receiving PTU and apelin at the same time; PT: receiving PTU and T4at the same time; PAT: receiving PTU, apelin and T4 at the same time.

In hypothyroid animals, HR significantly reduced in comparison with the control group (145±9.5 vs. 227±7.8 beat/min, p < 0.001, Figure 1). In addition, the four-week administration of apelin to normal rats increased their HR (270±11.6 beat/min). Administration of apelin with PTU could not change HR as opposed to the hypothyroid group (157±16.4 vs. 145±9.5 beat/min). However, the administration of T4 with PTU could significantly prevent HR reduction (180±4 vs. 145±9.5 beat/min, p < 0.05), but the co-administration of apelin and T4 with PTU was more effective in rising HR (201±4.3 vs. 145±9.5 beat/min, p < 0.001).

### QRS voltage

**Figure 2:**
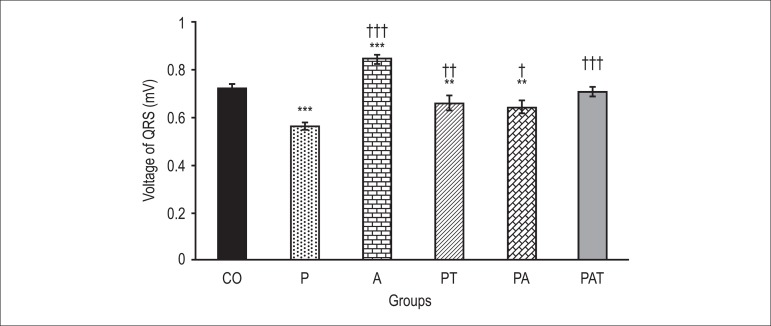
Comparison of QRS voltage in various groups. Data are expressed as mean±SEM. The analysis of data was done by one-way ANOVA followed by LSD test. **p < 0.01, ***p < 0.001 compared with the control; †p < 0.05, ††p < 0.01, †††p < 0.001 compared with the hypothyroid group. CO: control group; P: PTU-treated group; A: Intact animals group that received apelin; PA: receiving PTU and apelin at the same time; PT: receiving PTU and T4at the same time; PAT: receiving PTU, apelin and T4 at the same time.

The QRS voltage significantly decreased in the groups that received PTU as compared with the control group (0.563±0.015 vs. 0.72±0.02 mV, p < 0.001, Figure 2). However, apelin administration to normal animals increased QRS voltage significantly (0.844±0.022 mV, p < 0.001). Although the administration of apelin (0.646±0.026 mV, p < 0.05) or T4 (0.661±0.032 mV, p < 0.01) along with PTU could change QRS voltage, the co-administration of these three drugs together was more effective (0.708±0.02 mV, p < 0.001).

## Discussion

This study showed that the administration of apelin alone to normal rats or with T4 in PTU-induced hypothyroid rats decreases TSH, but increases T4 serum levels.

Taheri et al. have illustrated that intracerebroventricular administration of pyroglutamylated apelin-13 (10 nmol) decreased the TSH level although this reduction was not significant^21^. Pan et al^18^ have shown that T4 therapy decreased TSH level during 28 days after PTU-induced hypothyroidism. These findings suggest that apelin may have an effect on endocrine regulation and some hormones circulation. However, the regulatory effect of apelin on the thyroid axis and cell signaling calls for further study.

Comparing the results obtained from ECG in this study showed that HR and QRS voltage were reduced in the hypothyroid group as compared with those of the control group. On the other hand, the administration of apelin demonstrated an increasing effect on HR and QRS voltage in normal rats.

Thyroid hormones have an effective role in the regulation of the expression of some genes related to pacemaker cells; therefore, the loss of thyroid hormones causes a decrease in the sinoatrial node function^22,23^. Similarly to our finding, Joppet et al. have indicated that the intravenous apelin infusion in human increases HR and cardiac output^24^. Another study has shown that apelin has a positive chronotropic effect on myocardium via increasing cardiac excitability due to modulation of I_Na_ gating and amplitude^25^, which may be one of the reasons of the increase in HR by apelin. Our study showed that the co-administration of L-T4 and apelin in the PAT group prevents the decline in HR and QRS voltage in PTU-induced hypothyroid rats. Although the administration of each of these two drugs improves HR and QRS voltage, they significantly differed from the control group. It has been reported that the abnormality of ventricular systolic and diastolic functions in hypothyroidism was improved by L-T4^10^. It has also been identified that the variation of thyroid hormones could make a change in the expression of several gene proteins including: Ca^2+^-ATPase, phospholamban, myosin, beta-adrenergic receptors, adenylate cyclase, guanine-nucleotide-binding proteins, Na^+^/Ca^2+^ exchanger, Na^+^/K^+^ ATPase, and voltage gated-potassium channels^3^. The decrease in heart contraction in hypothyroidism is related to the decrease in sarcoplasmic reticulum Ca^2+^-ATPase gene expression and the increase in phospholamban^3^.

Berry et al^26^ have reported that apelin has an inotropic effect by increasing the cardiac output without changing the end-diastolic volume. Apelin peptides are among the most potent endogenous positive inotropic agents^27^. The inotropic effect of apelin mediated through G-protein coupled to APJ receptor activates protein kinase C, which affects Na^+^/H^+^ exchanger; however, this promotes inner cell alkalinization and sensitization of myofilaments to Ca2^+^. On the other hand, it affects Na^+^/Ca^2+^ exchanger and increases cytoplasmic Ca^2+^ ^11,14,24^. Wang et al^28^ have shown that L-T4 increases alpha-myosin heavy chain (αMHC) isoform gene expression, which improves the heart contraction potential. This suggests that the co-administration of L-T4 and apelin, probably regulates the gene expression of contraction proteins and increases the sensitivity of myofilaments to calcium^24^.

According to previous studies and the findings of this study, it is suggested that apelin may have a role in cardiac contractility by changing phospholipase C, protein kinase C, Na^+^/H^+^ exchanger and sarcolema Na^+^/Ca^2+^ exchanger gene expression in hypothyroid rats.

## Conclusion

In conclusion, although apelin increases cardiac voltage in the absence of thyroid hormone, this mechanism of apelin is more effective in the presence of the thyroid hormone.

## References

[1] McAninch EA, Bianco AC (2014). Thyroid hormone signaling in energy homeostasis and energy
metabolism. Ann N Y Acad Sci.

[2] Coceani M (2013). Heart disease in patients with thyroid dysfunction: hyperthyroidism,
hypothyroidism and beyond. Anadolu Kardiyol Derg.

[3] Klein I, Danzi S (2007). Thyroid disease and the heart. Circulation.

[4] Biondi B (2012). Mechanisms in endocrinology: heart failure and thyroid
dysfunction. Eur J Endocrinol.

[5] Celik A, Aytan P, Dursun H, Koc F, Ozbek K, Sagcan M (2011). Heart rate variability and heart rate turbulence in hypothyroidism
before and after treatment. Ann Noninvasive Electrocardiol.

[6] Rhee SS, Pearce EN (2011). Update: Systemic Diseases and the Cardiovascular System (II). The
endocrine system and the heart: a review. Rev Esp Cardiol.

[7] Galetta F, Franzoni F, Fallahi P, Tocchini L, Braccini L, Santoro G (2008). Changes in heart rate variability and QT dispersion in patients with
overt hypothyroidism. Eur J Endocrinol.

[8] Wald DA (2006). ECG Manifestations of selected metabolic and endocrine
disorders. Emerg Med Clin North Am.

[9] Ching GW, Franklyn JA, Stallard TJ, Daykin J, Sheppard MC, Gammage MD (1996). Cardiac hypertrophy as a result of long-term thyroxine therapy and
thyrotoxicosis. Heart.

[10] Gammage M, Franklyn J (1997). Hypothyroidism, thyroxine treatment, and the heart. Heart.

[11] Chandrasekaran B, Dar O, McDonagh T (2008). The role of apelin in cardiovascular function and heart
failure. Eur J Heart Fail.

[12] De Falco M, De Luca L, Onori N, Cavallotti I, Artigiano F, Esposito V (2002). Apelin expression in normal human tissues. In Vivo.

[13] Kursunluoglu-Akcilar R, Kilic-Toprak E, Kilic-Erkek O, Turgut S, Bor-Kucukatay M (2014). Apelin-induced hemorheological alterations in DOCA-salt hypertensive
rats. Clin Hemorheol Microcirc.

[14] Ladeiras-Lopes R, Ferreira-Martins J, Leite-Moreira AF (2008). The apelinergic system: the role played in human physiology and
pathology and potential therapeutic applications. Arq Bras Cardiol.

[15] Tatemoto K, Takayama K, Zou MX, Kumaki I, Zhang W, Kumano K (2001). The novel peptide apelin lowers blood pressure via a nitric
oxide-dependent mechanism. Regul Pept.

[16] Wu S, Gao Y, Dong X, Tan G, Li W, Lou Z (2013). Serum metabonomics coupled with Ingenuity Pathway Analysis
characterizes metabolic perturbations in response to hypothyroidism induced by
propylthiouracil in rats. J Pharm Biomed Anal.

[17] Falcão-Pires I, Gonçalves N, Henriques-Coelho T, Moreira-Gonçalves, Roncon-Albuquerque R Jr, Leite-Moreira AF (2009). Apelin decreases myocardial injury and improves right ventricular
function in monocrotaline-induced pulmonary hypertension. Am J Physiol Heart Circ Physiol.

[18] Pan T, Zhong M, Zhong X, Zhang Y, Zhu D (2013). Levothyroxine replacement therapy with vitamin E supplementation
prevents oxidative stress and cognitive deficit in experimental
hypothyroidism. Endocrine.

[19] Vetter R, Rehfeld U, Reissfelder C, Fechner H, Seppet E, Kreutz R (2011). Decreased cardiac SERCA2 expression, SR Ca uptake, and contractile
function in hypothyroidism are attenuated in SERCA2 overexpressing transgenic
rats. Am J Physiol Heart Circ Physiol.

[20] Rodriguez-Angulo H, Garcia O, Castillo E, Cardenas E, Marques J, Mijares A (2013). Etanercept induces low QRS voltage and autonomic dysfunction in mice
with experimental Chagas disease. Arq Bras Cardiol.

[21] Taheri S, Murphy K, Cohen M, Sujkovic E, Kennedy A, Dhillo W (2002). The effects of centrally administered apelin-13 on food intake, water
intake and pituitary hormone release in rats. Biochem Biophys Res Commun.

[22] Pachucki J, Burmeister LA, Larsen PR (1999). Thyroid hormone regulates hyperpolarization-activated cyclic
nucleotide-gated channel (HCN2) mRNA in the rat heart. Circ Res.

[23] Paslawska U, Noszczyk-Nowak A, Kungl K, Bioly K, Popiel J, Nicpon J (2006). Thyroid hormones concentrations and ECG picture in the
dog. Pol J Vet Sci.

[24] Japp AG, Cruden NL, Barnes G, van Gemeren N, Mathews J, Adamson J (2010). Acute cardiovascular effects of apelin in humans: potential role in
patients with chronic heart failure. Circulation.

[25] Chamberland C, Barajas-Martinez H, Haufe V, Fecteau MH, Delabre JF, Burashnikov A (2010). Modulation of canine cardiac sodium current by Apelin. J Mol Cell Cardiol.

[26] Berry MF, Pirolli TJ, Jayasankar V, Burdick J, Morine KJ, Gardner TJ (2004). Apelin has in vivo inotropic effects on normal and failing
hearts. Circulation.

[27] Maguire JJ, Kleinz MJ, Pitkin SL, Davenport AP (2009). [Pyr1]apelin-13 identified as the predominant apelin isoform in the
human heart: vasoactive mechanisms and inotropic action in disease. Hypertension.

[28] Wang YY, Jiao B, Guo WG, Che HL, Yu ZB (2010). Excessive thyroxine enhances susceptibility to apoptosis and decreases
contractility of cardiomyocytes. Mol Cell Endocrinol.

